# Deep Transcranial Magnetic Stimulation in Patients With Opioid Use Disorder: A Double‐Blind, Placebo‐Controlled Randomized Trial

**DOI:** 10.1111/adb.70057

**Published:** 2025-06-19

**Authors:** Soner Guldas, Selim Tumkaya, Bengu Yucens

**Affiliations:** ^1^ Department of Psychiatry Gaziantep State Hospital Gaziantep Turkey; ^2^ Faculty of Medicine, Department of Psychiatry Pamukkale University Denizli Turkey; ^3^ Faculty of Medicine, Department of Neuroscience Pamukkale University Denizli Turkey

**Keywords:** brain stimulation, heroin, opioid use disorder, transcranial magnetic stimulation

## Abstract

Opioid use disorder (OUD) is a chronic, relapsing brain disorder. The efficacy of brain stimulation methods in the treatment of OUD has been increasingly investigated. However, the efficacy of deep transcranial magnetic stimulation, namely, wide‐volume TMS, in the treatment of OUD has not been investigated. The aim of this randomized, double‐blind, sham‐controlled add‐on study was to evaluate the efficacy of wide‐volume TMS using a double‐cone coil in participants with OUD. A total of 55 OUD patients were recruited and randomized to receive either active or sham TMS. Active wide‐volume TMS treatment was applied to the left dorsolateral prefrontal cortex (DLPFC) in the active TMS group using a double‐cone coil, twice daily for 2 weeks, at a frequency of 10 Hz. Sham TMS was also applied to the same region in the placebo group using a placebo coil. Opioid Craving Visual Analogue Scale (OC‐VAS), Hamilton Depression Rating Scale (HDRS), Hamilton Anxiety Rating Scale (HARS) and Barratt Impulsiveness Scale‐11 (BIS‐11) were measured before treatment, at the end of treatment, and 2 months after treatment. A total of 21 patients from the active TMS group and 19 patients from the sham TMS group completed the study. Although the active TMS group exhibited more reduction in craving and a less pronounced increase in buprenorphine‐naloxone dose during the treatment period compared to the sham group, these differences did not reach statistical significance. This study suggests that while wide‐volume TMS using a double‐cone coil applied to the left DLPFC was well tolerated, it did not produce statistically significant improvements in craving, depression, anxiety, or impulsivity when compared to the sham treatment. However, the observed trends warrant further investigation with larger sample sizes and refined protocols.

****Trial Registration:** This trial was registered at ClinicalTrials.gov (NCT06081985):**

## Graphical abstract

This randomized controlled trial evaluated the efficacy of accelerated wide‐volume deep TMS using a double‐cone coil in patients with opioid use disorder. Although no statistically significant improvements were observed, the active group showed a trend toward reduced craving and stable buprenorphine‐naloxone doses compared to the sham group. The findings highlight the need for larger trials targeting alternative brain regions.

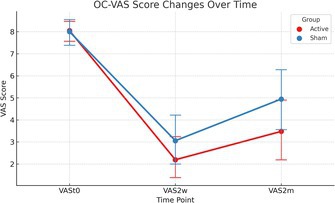

## 1 Introduction

Opioid use disorder (OUD) is characterized by persistent craving for opioid‐containing substances, continued use despite physical and/or psychological impairment, increased tolerance with use and the onset of withdrawal symptoms when opioids are stopped [1]. It has been reported that OUD affects more than 16 million people worldwide, and more than 120 000 deaths worldwide are attributed to opioids each year [2].

OUD is thought to begin with the activation of reward circuitry in the brain, which over time progresses to antireward circuitry leading to negative emotional states and relapses. Opioid use activates the mesolimbic reward system in the brain and causes the release of dopamine from neurons in the ventral tegmental area to the nucleus accumbens [3, 4]. While activation of the brain's reward system is the main reason for continued opioid use in the early stages of opioid use, compulsive opioid use occurs with the development of tolerance and addiction [3, 4]. Although opioid agonist treatments are used in the treatment of OUD, a significant proportion of people receiving these treatments have been found to continue using illicit opioids during treatment, and relapse is common after the tapering of opioid agonist treatment [5]. This has led to a search for new treatment modalities for OUD.

In recent years, rTMS treatment for OUD has emerged as a new area of research. TMS can modulate cortical excitability, alter the release of neurotransmitters and induce long‐lasting changes in neuronal excitability [6]. Previous studies have demonstrated the direct effects of rTMS on spontaneous and cue‐induced craving. Shen et al. [7] and Jin et al. [8] reported reduced craving scores after 5 and 7 rTMS sessions over the left dorsolateral prefrontal cortex (DLPFC), respectively. Liu et al. [9] found that 20 sessions of both 1‐ and 10‐Hz frequency rTMS over the left DLPFC reduced cue‐induced craving and that the reduction in craving remained significant at 60‐day follow‐up. However, this trial was not placebo‐controlled; it was a comparison with patients on a waiting list. Contrary to the results of these studies, there are also placebo‐controlled studies showing that rTMS is ineffective on craving. Sahlem et al. [10] reported no significant reduction in craving after a single rTMS session over the left DLPFC, and Tsai et al. [11] reported no change in heroin use and craving after a treatment that included 11 sessions of 15 Hz frequency rTMS over the left DLPFC in combination with methadone maintenance therapy. Despite the conflicting results, these studies suggest that the DLPFC may be a suitable target for TMS treatment in patients with OUD. The left DLPFC has been previously reported to play a role in executive functions and craving regulation, and stimulating this brain region may reduce craving by improving inhibitory control [12]. On the other hand, the conflicting results observed in the aforementioned studies may be due to the insufficient efficacy of TMS protocols that stimulate only small brain volumes with figure‐of‐eight coils [13]. An alternative approach that could enhance this efficacy is the application of deep TMS. To date, no study has investigated the efficacy of deep TMS in OUD patients. TMS protocols capable of affecting larger brain volumes than the figure‐of‐eight coil have been referred to in the literature as deep TMS. However, increasing the depth of a magnetic stimulus also increases its spatial spread [14]. Therefore, in this article, such protocols will be referred to as ‘wide‐volume TMS’ (wvTMS).

Although not yet FDA approved for substance use disorders (SUDs), a growing number of studies are investigating the efficacy of TMS for other SUDs, such as alcohol and smoking, with promising positive results [15, 16, 17]. These TMS studies targeted the DLPFC with H‐coil and applied high‐frequency stimulation. For instance, Addolorato et al. (2017) applied wvTMS with H‐coil bilaterally to the DLPFC for 12 sessions in patients with alcohol use disorder, reporting reductions in alcohol intake and changes in dopamine transporter availability, suggesting a neurobiological effect of wvTMS on the dopaminergic system [15]. Similarly, Girardi et al. (2015) demonstrated that add‐on 20 sessions of wvTMS to standard treatment significantly reduced alcohol craving and depressive symptoms in patients with alcohol use disorder comorbid with dysthymia [16]. Additionally, Rapinesi et al. (2016) found that high‐frequency wvTMS for 12 sessions over the bilateral DLPFC reduced cocaine craving, with effects persisting for several weeks post‐treatment [17].

On the other hand, it is also known that treatment compliance problems are prominent in patients with SUD [18]. In recent years, research on accelerated TMS protocols has gained momentum, as accelerated TMS treatments may be more effective in a shorter time frame, potentially improving patient compliance with treatment [19]. Although there are accelerated TMS studies in patients with cocaine use disorder [20, 21], to our knowledge, there is no study on the effectiveness of accelerated TMS protocols in OUD patients. Based on all these findings, this study aimed to investigate the effects of 20 sessions of 10 Hz TMS with a double‐cone coil applied twice daily to the left DLPFC on craving, impulsivity, depression and anxiety levels in OUD patients.

## 2 Methods

### 2.1 Participants

A total of 55 participants who met the diagnostic criteria for OUD according to the Diagnostic and Statistical Manual of Mental Disorders, Fifth Edition (DSM‐5) were recruited from the Alcohol and Drug Addiction Research, Treatment and Training Centre at the Faculty of Medicine, Pamukkale University. Each patient was initially interviewed and diagnosed by an experienced psychiatrist. Inclusion criteria were age between 18 and 65 years and abstinence from heroin for at least 2 weeks. Exclusion criteria included the following: (1) clinically significant psychiatric disorders such as bipolar disorder, psychotic disorders and neurocognitive disorders; (2) epilepsy; (3) unstable medical conditions; (4) pacemakers and metal implants; and (5) pregnancy.

**FIGURE 1:**
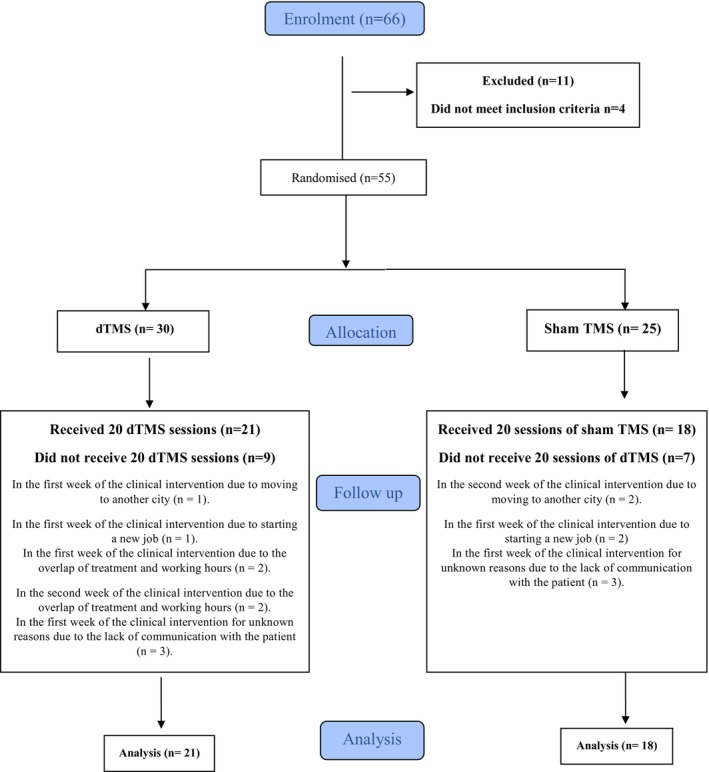
Consort diagram.

A power analysis was performed for the study, and it was found that the effect size obtained in the reference study [11] was large (*d* = 1.16). As a result of the power analysis performed, considering that a smaller effect size could also be obtained (d = 0.9), it was calculated that 80% power could be obtained at the 95% confidence level when at least 32 people (at least 16 people for each group) were included in the study. Considering that patients dropping out during the study would reduce the sample size and the power of the study, it was planned to recruit patients instead of patients dropping out. By this way, it was planned to increase the sample size to account for dropout. A randomization list was generated for the active and sham groups according to the 1:1 ratio in the Research Randomizer program [22], and patients were allocated to the active and sham groups by the TMS technician according to this list. The consort diagram in Figure 1 shows the process of participant participation and completion of the study. The TMS technician replaced patients who had dropped out with the first newly recruited patient and then continued to allocate patients to the groups according to the randomization list. Due to the expiration of the project budget, the last patient who should have been enrolled in the sham group could not be enrolled. In addition, the number of patients in the active and sham groups could not be balanced because two patients who dropped out of the active group after the enrolment period could not be replaced by new patients. Thus, by the end of the project budget, 16 of the 55 participants had not completed 20 sessions of active/sham TMS. Of the 39 participants who completed 20 sessions of TMS treatment, 21 were in the active group, and 18 were in the sham group. Of the 16 patients who did not complete the study, 9 received active and 7 received sham TMS.

The research assistant who assessed the participants and the clinicians who administered the buprenorphine‐naloxone doses to the participants were blinded to active and sham groups. It was the research assistant who assessed the patients and administered the scales before the TMS treatment began. The participants did not know which group they would be assigned to. Only the TMS technician knew whether the patients were in the active or the sham group. The TMS technician administered the cue induction before each session. Buprenorphine‐naloxone treatment was continued throughout the study. The dose of buprenorphine‐naloxone could be tapered during the study. Buprenorphine‐naloxone doses were adjusted based on the clinician's assessment, the patient's subjective withdrawal symptoms and treatment guidelines. There were no restrictions on the medications used during the study, and outcomes were observed and recorded. All but three patients (two patients in the active group and one patient in the placebo group) were receiving buprenorphine‐naloxone treatment at inclusion.

The Institutional Review Board at Pamukkale University Faculty of Medicine approved the research protocol. This research was funded by Scientific Research and Projects (grant numbers: 2022HZDP025‐2022HZDP026). The funding body had no role in the study design, data collection, analysis, interpretation or manuscript preparation. The study was conducted according to the ethical standards of the Helsinki Declaration of 1964. The procedures were fully explained to each participant before they were asked to sign an informed consent form. This study is part of the OUD Clinical Trials Analysis (Trial registration: NCT06081985)

### 2.2 TMS Procedure

In this study, wvTMS was applied to the group receiving active TMS with a double‐cone coil compatible with the Neuro‐MS/D device. The sham TMS application protocol was used with a custom‐designed sham TMS coil called AFEC‐03‐100‐P‐P‐Placebo, which mimics the active coil in terms of acoustics and scalp sensation and produces comparable activation of facial muscles without stimulating the brain.

The magnetic magnitude at which the contralateral abductor pollicis brevis muscle was stimulated by a single magnetic pulse was used as the motor threshold. The location of the left dorsolateral prefrontal region was 5‐cm anterior to the point on the scalp where the contralateral abductor pollicis brevis muscle responded. In a randomized double‐blind manner, each patient in the active group received a total of 20 wvTMS sessions (twice daily for 2 weeks): 75 trains per session, 40 pulses per train at a frequency of 10 Hz and an intertrain interval of 11 s, 3000 pulses/day, 110% of the motor threshold. The motor threshold was measured again at the beginning of each TMS session. During the 2‐week treatment phase, participants received two wvTMS sessions per day with an inter‐session interval of approximately 4 h. Sham stimulation consisted of TMS sessions with a placebo coil. Both patient groups and investigators were blinded to the type of stimulation. Adverse events were self‐reported and recorded.

In addition, just before the clinical intervention sessions, participants were shown a 5‐min visual slideshow of real‐life photographs of opioid use (including both injection and inhalation procedures). They were asked to suppress the resulting craving during the clinical intervention sessions. Relevant visual cues were obtained from the International Affective Picture System (IAPS).

Participants were followed up for 2 months after the wvTMS application, and urine drug test results were examined for patients who continued their routine treatment at the outpatient clinic of the Alcohol and Drug Addiction Research, Treatment, and Training Centre. Urine tests were conducted at baseline and at follow‐up monthly visits.

### 2.3 Measurements

Participants were interviewed by a trained investigator blinded to the group using a detailed questionnaire including demographics and current and past substance use. The Opioid Craving‐Visual Analogue Scale (OC‐VAS), Hamilton Depression Rating Scale (HDRS), Hamilton Anxiety Rating Scale (HARS) and Barratt Impulsivity Scale (BIS‐11) were administered to the patients before, at the end of the treatment (Week 2) and at the 2‐month follow‐up.

### 2.4 The Opioid Craving‐Visual Analogue Scale (OC‐VAS)

The OC‐VAS [23] was administered to participants before treatment, at the end of treatment (2 weeks) and 2 months after treatment. Participants were asked to rate the severity of their craving for opioids on a 10‐cm line (0 = no craving and 10 = strongest craving ever). Spontaneous craving was assessed, not cue‐induced craving. The psychometric evaluation of the OC‐VAS has been studied in a large population with OUD. The ability of the psychometric assessment of the OC‐VAS to predict opioid use and its correlation with clinician‐reported global measures supports the comprehensive assessment of patients with OUD with this scale and the evaluation of the effectiveness of OUD treatments [23].

### 2.5 HDRS

The HDRS is a 17‐item self‐report Likert scale and was used to assess the severity of current depressive symptoms [24]. The validity and reliability study of the Turkish version of the HDRS were conducted by Akdemir et al. in 1996 [25].

### 2.6 HARS

The HARS measures the severity of anxiety symptoms [26]. Yazıcı et al. (2008) conducted a validity and reliability study of the Turkish version of the HARS [27].

### 2.7 BIS‐11

BIS‐11 is a self‐report 4‐point Likert scale used to assess the severity of impulsive traits [28]. Higher scores on the BIS‐11 reflect higher levels of impulsivity. The validity and reliability study of the Turkish version was conducted by Güleç et al. [29].

Doses of buprenorphine‐naloxone use were also recorded during the treatment and follow‐up period. Although not a primary outcome of the study, urine opioid analysis results, which were routinely performed during patients' treatment in the clinic, were obtained from hospital records. The study analysed urine opioid test results from the start of TMS treatment to the end of the 2‐month follow‐up period, and patients with at least one opioid‐positive result were considered to be opioid users.

### 2.8 Statistical Analysis

In the statistical analysis of the study, SPSS (Statistical Package for Social Sciences) version 22.0, a Windows package program, was used to analyse the data. The Chi‐square test was used to compare categorical variables between two groups. To compare continuous variables between the two groups, we checked whether they fit the normal distribution. For independent variables, the Mann–Whitney *U*‐test was used to determine whether continuous variables that did not fit the normal distribution differed between groups, and the Student *t*‐test was used to compare continuous variables that did fit the normal distribution. As there were repeated assessments, the Generalized Estimating Equations (GEE) analyses were used to compare changes in dependent variables over time between the active and sham groups. Buprenorphine‐naloxone dose, age, sex and duration of opioid use were controlled for when evaluating the group*time interaction for craving, depression, anxiety and impulsivity levels. Pair‐wise comparisons between the active and sham groups at each time point were also analysed with Student *t*‐test, and effect sizes were calculated using Cohen's *d*. Statistical significance was accepted as *p* < 0.05 for the tests.

An ITT analysis was also performed, including all patients who participated in the randomization phase (supplement). For this dataset, a comparison of the sociodemographic characteristics of the groups (Table S1), the medications used and their daily doses, OC‐VAS, HDRS, HARS, BIS‐11 scores, and buprenorphine‐naloxone doses (Table S2) is presented in the Supporting Information Appendix. A total of 55 patients were included in the ITT sample. Of these, 16 patients dropped out, and therefore, only baseline values were available for these patients. When analysed with the MCAR test, the missing data were found to be completely random [*χ* ^2^ = (555, *N* = 55) = 0, *p* = 1.000]. Missing data for these patients were imputed by maximum likelihood estimation using the expectation maximization algorithm. The results of the analyses performed after imputation were not different from those performed before imputation.

## 3 Results

The demographic and clinical characteristics of the groups are shown in Table 1. There were no statistically significant differences between the active and sham groups concerning age, sex, educational status, marital status, duration of opioid use, average buprenorphine‐naloxone dose and duration of buprenorphine‐naloxone use. Fluoxetine‐equivalent doses of antidepressants and olanzapine‐equivalent doses of antipsychotics were calculated. The mean fluoxetine‐equivalent dose of the active group was 11.01 ± 16.01, and the mean olanzapine‐equivalent dose was 2.92 ± 5.42; the mean fluoxetine‐equivalent dose of the sham group was 16.10 ± 17.65, and the mean olanzapine‐equivalent dose was 2.69 ± 2.78. When the active and sham groups were compared in terms of pretreatment fluoxetine (*Z* = −0.879, *p* = 0.379), olanzapine (*Z* = −0.729, *p* = 0.476) and buprenorphine‐naloxone (*Z* = −0.296, *p* = 0.767) equivalent doses; no significant difference was found.

**TABLE 1:** Comparison of demographic characteristics of active and sham groups.

		Active (*n* = 21)	Sham (*n* = 18)	*t/X* ^2^	*p*
Age	Years	25.38 ± 3.46	25.83 ± 3.29	0.416	0.680^a^
Sex	Male	19 (90.5%)	14 (77.8%)	1.201	0.387
	Female	2 (9.5%)	4 (22.2%)		
Marital Status	Married	5 (23.8%)	4 (22.2%)	0.014	1.000
	Single	16 (76.2%)	14 (77.8%)		
Education	Primary school	15 (71.4%)	11 (61.1%)	0.464	0.520
	Secondary school and above	6 (28.6%)	7 (38.9%)		
Duration of opioid use	< 5 years	3 (14.3%)	1 (5.6%)	2.125	0.609
	> 5 years	18 (85.7%)	17 (94.4%)		
Duration of opioid use	Years	6.04 ± 2.61	7.50 ± 2.45	1.777	0.084^a^
Average buprenorphine‐naloxone dose	mg/day	9.27 ± 5.10	10.46 ± 5.16	−0.729	0.466^b^
Duration of buprenorphine‐naloxone use	Days	108 ± 197.56	96.58 ± 175.61	−0.635	0.525^b^
Comorbid psychiatric disorders	Generalized anxiety disorder	7 (33.3%)	12 (66.7%)	4.311	0.038
	Panic disorder	3 (14.3%)	0 (0%)	2.786	0.095
	Major depressive disorder	18 (85.7%)	14 (77.8%)	0.415	0.520
	Obsessive‐compulsive disorder	0 (0%)	1 (5.6%)	1.197	0.274

^a^ Student *t*‐test.

^b^ Mann–Whitney *U*‐test.

The majority of participants were on stable buprenorphine‐naloxone maintenance doses: 15 patients on a stable dose (83.3%), 0 patient on a decreased dose (0%) and 3 patients on an increased dose (16.7%) in the sham group and 13 patients on a stable dose (61.9%), 4 patients on a decreased dose (19%) and 4 patients on an increased dose (19%) in the active group. There was no statistically significant difference between sham and active groups (chi‐square = 4.079, *p* = 0.130).

There were five patients (23.8%) in remission in the active group and three patients (16.7%) in the sham group. There was no significant difference between the groups in terms of the number of patients in remission (Fischer's exact test *p*‐value = 0.702). Patients who did not receive buprenorphine‐naloxone treatment were in remission in both groups.

Comorbid substance use of participants was evaluated according to the results of urine analyses performed in the last 3 months. The results of urine analyses showed that four patients (22.2%) in the sham group and four patients (19%) in the active group were positive for substances other than opiates. No statistically significant difference was found between the active and sham groups in terms of other substance use (Fischer's exact test *p*‐value = 1.000).

The frequency of comorbid psychiatric disorders was compared between the active and sham groups. The number of patients with generalized anxiety disorder was significantly higher in the sham group than in the active group. No significant difference was found between the two groups in terms of the frequency of panic disorder, major depressive disorder and obsessive‐compulsive disorder (Table 1).

During the treatment period, nine (30%) patients in the active group and seven (28%) in the sham group dropped out. When comparing dropout rates, no significant difference was found between the active and sham groups (*X* ^2^ = 0.026, *p* = 1.000).

In the primary results of the study, when the mean OC‐VAS scores were taken as the dependent variable, with age, sex, duration of opioid use and buprenorphine‐naloxone dose as covariates in the Generalized Estimated Equations method, the interaction between group and time was not statistically significant (Table 2). During the follow‐up period, only two patients had positive urine opioid analysis tests, and both patients were in the active group. Urine opioid test results of four patients from the active group and one patient from the placebo group were missing. There was no statistically significant difference in urine opioid test results between the two groups (*X* ^2^ = 2.125, *p* = 0.145).

**TABLE 2:** Comparison of the change in OC‐VAS, HDRS, HARS and BIS‐11 scores at baseline, at the end of treatment, and in month two between active and sham groups.

		Wald *X* ^2^	*B*	*df*	*p* a
OC‐VAS	Group	3.104	1.468	1	0.078
	Time	158.292	4.571	2	< 0.001
	Group*time	2.665	−1.516	2	0.264
HDRS	Group	1.423	1.937	1	0.233
	Time	78.425	11.048	2	< 0.001
	Group*time	0.542	0.508	2	0.763
HARS	Group	3.614	2.754	1	0.057
	Time	54.453	9.714	2	< 0.001
	Group*time	0.528	2.119	2	0.768
BIS‐11	Group	1.318	4.476	1	0.251
	Time	28.186	12.429	2	< 0.001
	Group*time	1.080	−0.651	2	0.583

Abbreviations: BIS‐11, Barrat Impulsiveness Scale‐11; HARS, Hamilton Anxiety Rating Scale; HDRS, Hamilton Depression Rating Scale; OC‐VAS, Opioid Craving Visual Analogue Scale.

^a^ Age, sex, duration of opioid use and buprenorphine‐naloxone dose were taken as covariates.

**FIGURE 2:**
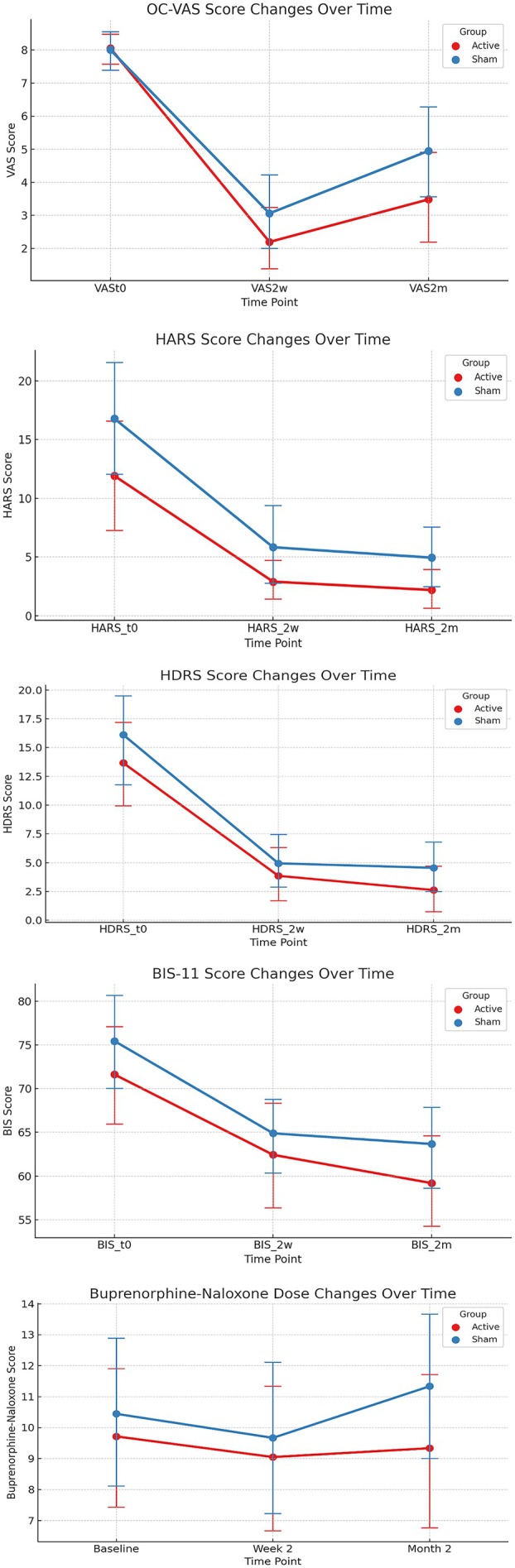
The change graphs of OC‐VAS, HARS, HDRS, BIS‐11 and buprenorphine‐naloxone dose from the baseline to the end of the 2‐month follow‐up.

In the secondary outcomes, when HDRS, HARS and BIS‐11 scores were taken as the dependent variables and age, sex, duration of opioid use and buprenorphine‐naloxone dose were taken as covariates in the GEE method, the interaction between group and time was not statistically significant (Table 2). A multicollinearity analysis was conducted using variance inflation factor (VIF) values for the main covariates included in the regression model (age, sex, duration of opioid use and mean buprenorphine‐naloxone dose). All VIF values were below 2, indicating that there was no significant multicollinearity among the predictors. Additionally, to evaluate model parsimony and collinearity risk, a reduced GEE model was compared to the full model including all covariates. The reduced model, including only buprenorphine‐naloxone dose, showed similar goodness‐of‐fit (QICC = 627.3) to the full model, indicating no significant benefit of the additional covariates in this sample. The change graphs of OC‐VAS, HARS, HDRS, BISS‐11 and buprenorphine‐naloxone dose from the baseline to the end of the 2‐month follow‐up are shown in Figure 2.

Cohen's *d* effect sizes were calculated for group differences in OC‐VAS, HDRS, HARS and BIS‐11 scores at each time point (Table 3). Among these results, it is seen that the effect sizes show an increasing trend from baseline to the second month for OC‐VAS.

**TABLE 3:** Summary of between‐group differences and effect sizes for clinical outcome measures at each time point.

		Mean difference	*t*	*p*	Cohen's *d*
OC‐VAS	Baseline	−0.05	−0.122	0.904	−0.04
	Week 2	0.87	1.158	0.254	0.37
	Month 2	1.47	1.465	0.151	0.47
HDRS	Baseline	2.44	0.892	0.378	0.29
	Week 2	1.09	0.622	0.538	0.20
	Month 2	1.94	1.276	0.210	0.41
HARS	Baseline	4.87	1.430	0.162	0.46
	Week 2	2.93	1.570	0.125	0.51
	Month 2	2.75	1.710	0.095	0.55
BIS‐11	Baseline	3.83	0.904	0.372	0.29
	Week 2	2.46	0.642	0.525	0.21
	Month 2	4.48	1.135	0.263	0.37

There were 14 cases of mild headache in the active group (46.7%) and 4 cases in the sham group (16%). This adverse event was significantly more common in the active group than in the sham group (*X* ^2^ = 5.825, *p* = 0.016). The duration of this side effect was generally 1 to 2 days. No serious adverse events were observed or reported by the subjects.

## 4 Discussion

This is the first double‐blind, randomized controlled trial to evaluate the efficacy of wvTMS on the left DLPFC in OUD patients. Accelerated wvTMS (2 daily sessions for 5 days/week), for a total of 20 sessions and 3000 pulses in each session, was applied to the left DLPFC at a frequency of 10 Hz using a double‐cone coil. No serious adverse events were observed or reported, indicating the safety of the wvTMS treatment. Patients with OUD were followed up for 2 months after treatment, and treatment efficacy was also assessed longitudinally. Although an increase in effect size was observed in craving scores from baseline to Month 2, this increase did not reach statistical significance, which was the study's primary outcome. Similarly, no statistically significant differences were found in depression, anxiety and impulsivity, which were the secondary outcomes, between the active and sham groups.

The DLPFC is one of the most targeted brain regions in TMS research on SUDs because of its central role in executive functions, including the control of substance intake, its hypoactivity in SUDs and its close association with neural circuits associated with substance use [12, 30]. It has been suggested that stimulation of the DLPFC with rTMS may lead to a reduction in the craving score by altering cortical regions associated with behavioural inhibition or decision‐making and by increasing synaptic plasticity [31]. However, previous studies in OUD patients using figure‐of‐eight coil rTMS in the DLPFC have yielded conflicting results. In this study, in which wvTMS was applied to target the DLPFC, the observed effectiveness did not reach statistical significance in terms of craving. Possible reasons for this lack of sufficient effectiveness may include the region of application. Other regions implicated in the pathophysiology of SUDs are the basal ganglia and the amygdala. It has been suggested that the basal ganglia are involved in the control of the rewarding or pleasurable effects of substance use and recurrent substance use, while the amygdala is associated with feelings of anxiety, fear and irritability that accompany substance withdrawal [12, 32]. Although the DLPFC is a region associated with executive function and control of impulsive behaviour, since craving is a symptom associated with the basal ganglia, wvTMS applied to the DLPFC may not be compelling enough on craving. A previous neuroimaging study found that the dorsal anterior cingulate cortex is biochemically and physiologically abnormal in patients with long‐term OUD on buprenorphine, requiring increased involvement of the frontoparietal and cerebellar behavioural regulatory network to achieve normal levels of task performance/behavioural control [33]. The fact that OUD patients included in this study were also using buprenorphine‐naloxone may have affected the effectiveness of wvTMS applied to the DLPFC. There is a need for studies investigating the effectiveness of vwTMS in reducing cravings when applied to other brain regions.

Coil design is also an important factor that can affect the results of TMS studies. Previous studies with wvTMS have found significant effects such as reduced craving and substance use, but these studies used the H‐coil [15, 16, 17]. The H‐coil and double‐cone coil are two types of TMS coils used for neuromodulation, each with distinct characteristics in terms of stimulation depth and field distribution. Considering that the H‐coil targets deeper subcortical regions in a wider area, whereas the double‐cone coil targets areas such as the DLPFC at a depth of 3–4 cm in a more focused manner [14], the H‐coil may provide greater efficacy due to its capacity to stimulate deeper subcortical areas such as ACC or nucleus accumbens; however, since the aim of this study was to target the DLPFC, a relatively superficial region, the double‐cone coil can be considered a suitable and adequate option. With the double‐cone coil, deep structures cannot be stimulated directly from the point where the DLPFC is targeted, but it is predicted that they can be modulated through functional connections.

In this study, the accuracy of DLPFC targeting using the 5‐cm rule may represent another important factor influencing the outcomes. The “5‐cm rule” is a commonly used but imprecise method for targeting the DLPFC in TMS studies. This method involves measuring 5‐cm anterior to the motor cortex hotspot along the scalp in a parasagittal line to approximate the DLPFC location. However, there is a lack of direct neuroanatomical targeting in this method [34]. Since the 5‐cm rule relies on external scalp measurements rather than neuroimaging, it does not guarantee stimulation of functionally relevant DLPFC areas implicated in cognitive or clinical outcomes. Neuronavigational systems provide significantly better accuracy than the 5‐cm rule, reducing variability in stimulation effects [35]. Further studies with neuronavigational systems are needed to replicate the findings of this study.

Compared to previous rTMS studies [7, 11, 36] conducted in patients with OUD, this study delivered a higher amount of stimulation, with 3000 pulses per session over a total of 20 sessions. Furthermore, while previous studies implemented stimulation protocols over a 2‐week period, this study applied an accelerated protocol (twice daily, 5 days per week). Studies have shown that a greater number of sessions can enhance long‐term neuromodulation effects [37]; however, increasing the number of sessions may also negatively affect treatment adherence. To balance these factors, the treatment duration was shortened by implementing an accelerated protocol, allowing for a higher number of sessions within a shorter timeframe. With this feature, this is the first study in which accelerated vwTMS was applied in OUD patients. Despite the accelerated protocol and a sufficient total number of sessions and pulses relative to other studies, another possible reason for the lack of favourable results may be that patients were simultaneously receiving buprenorphine‐naloxone treatment during the administration of wvTMS. Since buprenorphine is a partial opioid agonist, it may have affected the level of craving for opioids. Although the difference in buprenorphine‐naloxone doses between groups was not statistically significant at the 2‐month time point, a numerical increase was observed in the sham group compared to baseline, while the active group remained more stable. This pattern may reflect clinical adjustments made in response to perceived symptom worsening, craving, or tolerability in the sham group. The fact that OUD patients included in this study were also using buprenorphine‐naloxone may have masked the effectiveness of wvTMS, and future studies with more controlled medication management protocols and larger sample sizes may help clarify whether this observed increase is clinically meaningful or coincidental.

This study found that depression, anxiety and impulsivity scores, which were assessed as secondary outcomes, did not differ from the placebo group at the end of treatment and follow‐up. However, when rTMS was applied to the DLPFC, Tsai et al. and Lin et al. showed that depression and anxiety symptoms were significantly reduced in patients with methamphetamine and OUD [11, 38]. Gong et al. (2023) showed that iTBS to the left DLPFC was effective in reducing depression and anxiety levels in OUD patients receiving methadone maintenance treatment, but it was not shown to reduce impulsivity [39]. When the effect sizes were evaluated, although a mild effect trend was observed in depression, anxiety and impulsivity, these differences did not reach statistical significance. The limited sample size may have prevented the provision of sufficient statistical power, especially for secondary results. These parameters need to be tested by new studies with high‐quality methodology.

This study has some limitations. First, the small sample size may have caused a Type 2 error. This may have caused the apparent effects not to reach statistical significance. Furthermore, the ‘5‐cm method’ for targeting the DLPFC does not account for individual differences, which may result in a lack of neuroanatomical targeting. This limitation emphasizes the necessity of using neuronavigational systems in future studies. Another limitation of the study is that the quality of blinding methods was not tested. There is evidence that medical devices in general can induce a strong placebo response [40]. Outside of drug trials, it can be difficult to ensure that participants remain blinded. This is also the case for TMS research in SUDs. Successful blinding of participants to the intervention, that is, active or sham treatment, is essential to reduce the impacts of bias on treatment outcomes [41]. In the current study, wvTMS treatment was administered in addition to buprenorphine‐naloxone treatment, and there were also changes in buprenorphine‐naloxone doses during the treatment for naturalistic observation. The fact that patients' buprenorphine‐naloxone doses were increased when they had cravings may have caused the effectiveness of active TMS to be underestimated. Although the dose change was limited and the effect of the dose change was controlled by including the dose change as a covariate in statistical analyses, it may have affected the results of the OC‐VAS change. Thus, concurrent buprenorphine‐naloxone treatment may have attenuated the therapeutic effects of wvTMS. This study focused primarily on wvTMS as an adjunctive treatment to buprenorphine‐naloxone pharmacotherapy, and none of the patients received psychotherapy during the research. However, some participants did receive psychotropic medication. Although there was no significant difference between the active and sham groups when comparing the doses of medication used, the use of these medications may have influenced the results. Therefore, the effect of these factors should be taken into account when evaluating the negative results. In addition, the 2‐month follow‐up period can be considered relatively short and may have prevented the evaluation of the persistence of the effect after the end of treatment.

In conclusion, in this study, the craving scores of the OUD patients who received wide‐volume TMS decreased more compared to the sham group, and the sham group demonstrated a more pronounced increase in the need for buprenorphine‐naloxone during the treatment period compared to the active group. However, these differences did not reach statistical significance. Studies with larger sample sizes targeting different brain regions under neuronavigation guidance, using different coils, designed with accelerated protocols, will contribute to research in this field. Future studies should also explore the combinations with behavioural or pharmacological interventions to better understand the role of brain stimulation in treating OUD.

## Author Contributions

**Soner Guldas**: conceptualization, data acquisition, writing – original draft. **Bengu Yucens**: conceptualization, formal analysis, supervision, writing – original draft, writing – review and editing. **Selim Tumkaya**: conceptualization, formal analysis, supervision, writing – review and editing.

## Ethics Statement

This study was conducted in accordance with the ethical principles outlined in the Declaration of Helsinki and adhered to all relevant institutional and international research ethics and integrity guidelines. Ethical approval for this study was obtained from the Pamukkale University Ethics Committee, reference number (49201).

## Consent

All participants provided written informed consent prior to their inclusion in the study.

## Conflicts of Interest

The authors declare no conflicts of interest.

## Permission to Reproduce Material

Permission to reproduce material from other sources has been obtained where applicable.

## Supporting information


**Table S1.** Comparison of demographic characteristics of active and sham groups.
**Table S2.** Comparison of the change in OC‐VAS, HDRS, HARS, and BIS‐11 scores at baseline, at the end of treatment, and in month two between active and sham groups.

## Data Availability

The data supporting the findings of this study are available from the corresponding author upon reasonable request.
