# Supplementary material for: Deep Transcranial Magnetic Stimulation in Patients With Opioid Use Disorder: A Double‐Blind, Placebo‐Controlled Randomized Trial
Source: Addict Biol. 2025 Jun 19;30(6):e70057. doi: 10.1111/adb.70057 (PMC12178270; doi:10.1111/adb.70057)
Supplement: Supplementary file 1 — Table S1. Comparison of demographic characteristics of active and sham groups. Table S2. Comparison of the change in OC‐VAS, HDRS, HARS, and BIS‐11 scores at baseline, at the end of treatment, and in month two between active and sham groups. [file ADB-30-e70057-s001.docx]

**Table S1 Comparison of demographic characteristics of active and sham groups**

|  |  |  | Active (n=30) | Placebo (n=25) | t / X^2^ | p |
| --- | --- | --- | --- | --- | --- | --- |
| Age |  | years | 26.66 ± 4.96 | 26.40 ± 4.27 | -0.211 | 0.834* |
| Sex |  | male | 27 (90%) | 20 (80%) | 1.097 | 0.295 |
|  |  | female | 3 (10%) | 5 (20%) |  |  |
| Marital Status |  | married | 8 (26.7%) | 5 (20%) | 0.336 | 0.562 |
|  |  | single | 22 (73.3%) | 20 (80%) |  |  |
| Education |  | Primary school | 24 (80%) | 17 (68%) | 1.035 | 0.309 |
|  |  | high school and above | 6 (20%) | 8 (32%) |  |  |
| Duration of opioid use |  | <5 years | 5 (16.7%) | 4 (16.0%) | 0.004 | 0.947 |
|  |  | >5 years | 25 (83.3%) | 21 (84.0%) |  |  |
| Duration of opioid use |  | years | 6.33 ± 2.59 | 6.84 ± 2.76 | 0.701 | 0.487* |

Student t-test*

The mean fluoxetine equivalent dose of the active group was 13.41 ± 19.32, olanzapine equivalent dose was 2.72 ± 4.75, buprenorphine-naloxone equivalent dose was 8.26 ± 5.24; the mean fluoxetine equivalent dose of the sham group was 14.57 ± 16.85, olanzapine equivalent dose was 2.51 ± 2.97, buprenorphine-naloxone equivalent dose was 9.20 ± 5.44. When the active and sham groups were compared in terms of pre-treatment fluoxetine (Z=-0.536, p=0.592), olanzapine (Z=-0.439, p=0.661) equivalent dose averages and buprenorphine-naloxone dose averages (Z=-0.669, p=0.503), no significant difference was found.

**Table S2. Comparison of the change in OC-VAS, HDRS, HARS, and BIS-11 scores at baseline, at the end of treatment, and in month two between active and sham groups**

|  |  | Wald X^2^ | B | df | p* |
| --- | --- | --- | --- | --- | --- |
| OC-VAS | group | 3.116 | 1.039 | 1 | 0.078 |
|  | time | 132.121 | 3.488 | 2 | <0.001 |
|  | group*time | 0.966 | -0.845 | 2 | 0.617 |
| HDRS | group | 0.393 | 1.376 | 1 | 0.531 |
|  | time | 60.790 | 9.910 | 2 | <0.001 |
|  | group*time | 0.674 | -1.216 | 2 | 0.714 |
| HARS | group | 2.163 | 1.964 | 1 | 0.141 |
|  | time | 48.312 | 8.825 | 2 | <0.001 |
|  | group*time | 0.035 | 0.396 | 2 | 0.983 |
| BIS-11 | group | 1.536 | 3.213 | 1 | 0.215 |
|  | time | 23.372 | 8.484 | 2 | <0.001 |
|  | group*time | 1.211 | 0.607 | 2 | 0.546 |

*****Generalized Estimated Equation Analysis; age, sex, duration of opioid use, and buprenorphine-naloxone dose were taken as covariates.
